# Filariæ in the Blood of a Living Dog

**Published:** 1844-01-13

**Authors:** 


### FILARIÆ IN THE BLOOD OF A LIVING DOG.

MM. Gruby and Delafond communicated to the Academy of Sciences the discovery of the entozoa circulating in the blood of a strong and healthy dog. Physiologists have for a long time been aware of the presence of certain entozoa in the blood of reptiles and fishes, but this is the first instance in which they have been detected in the blood of a *living* mammal. It is of high importance to physiology, pathology, and natural history, to show not only the existence of worms in the blood, but also their circulation in this fluid, in the animals which come near to man in the scale of organization. These entozoa have a diameter of 0.003 millimetre, and a length of 0.25 millimetre. These bodies are transparent and colourless. Anterior extremity is obtuse—posterior or caudal extremity is terminated by a very slender filament. At the anterior part may be observed a little round depression, 0.005 millimetre long, which may be considered as the buccal fissure. Their motions are very active. Their life has been prolonged ten days after the blood has been taken from the vessels, and exposed to a temperature of 15° centigrade, or 59° Fahrenheit. They swim among the globules of the blood with great vivacity, exercising an undulating movement. MM. Gruby and Delafond found them in the blood taken from the coccygeal arteries, external jugular veins, capillaries of the conjunctivæ, mucous membrane, skin, muscles, and every where this liquid was found to contain them. The urine and other excrementitial matters did not contain them. The diameter of these entozoa being less than that of the blood corpuscles, enabled them to circulate through the capillary bloodvessels.—*London Medical Gazette, from Comptes Rendus.*

